# Convallaria Majalis

**Published:** 1884-06-20

**Authors:** 


**Convallaria Majalis.**—Convallaria majalis is not as perfectly safe as some have believed. Dr. George Herschell relates in the Lancet the case of a man, apparently healthy, who had an irregular pulse following worry and overwork two years ago. The patient had been taking digitalis, but this was discontinued, and, after an interval of a month or two, tincture of convallaria was ordered in five minim doses three times a day. After a few doses he was obliged to stop its use on account of its remarkable effects. Almost immediately after taking a dose the pulse became nearly imperceptible at the wrist, and there was a sense of oppression over the sternum, nausea, cold feet, vertigo, flatulence, and a feeling of utter prostration. These symptoms lasted two hours, but came on again at each repetition of the dose.—*Weekly Medical Review*, Dec. 1, 1883.

